# Incident Hepatocellular Carcinoma Risk in Patients Treated with a Sulfonylurea: A Nationwide, Nested, Case-Control Study

**DOI:** 10.1038/s41598-019-44447-1

**Published:** 2019-06-12

**Authors:** Ji-Yeon Lee, Suk-Yong Jang, Chung Mo Nam, Eun Seok Kang

**Affiliations:** 1 Department of Internal Medicine, Yonsei University College of Medicine, Seoul, Republic of Korea; 2 Department of Preventive Medicine, Eulgi University College of Medicine, Daejeon, Republic of Korea; 3 Department of Preventive Medicine, Yonsei University College of Medicine, Seoul, Republic of Korea

**Keywords:** Epidemiology, Risk factors

## Abstract

Several studies have shown that the use of sulfonylureas in patients with type 2 diabetes mellitus (T2DM) is associated with a higher risk of hepatocellular carcinoma (HCC). In this study, we investigated the effects of individual sulfonylureas on HCC development using the National Health Insurance Service-National Sample Cohort in South Korea. Among 47,738 subjects aged 40 years or older who had newly diagnosed with diabetes, 241 incident HCC cases and 1205 matched controls were identified. Adjusted odds ratios (ORs) as estimates of the relative risk of HCC were calculated using logistic regression analysis. Compared to patients never treated with a sulfonylurea, those treated with a sulfonylurea had a 1.7-fold increased risk of HCC development. Of the different types of sulfonylureas, the exclusive use of glimepiride was associated with a significantly elevated risk of HCC (OR = 1.89, 95% CI = 1.02–3.47) compared to those who were never treated with sulfonylureas. No significant associations were observed between exclusive gliclazide use and incident HCC (OR = 2.04, 95% CI = 0.75–5.52). In conclusion, the association between the use of sulfonylureas and risk of HCC was different according to the type of sulfonylurea, in patients with new-onset T2DM. Further prospective studies are warranted to confirm these results and translate them into clinical practice.

## Introduction

Hepatocellular carcinoma (HCC) is one of the most common cancers worldwide and is associated with a high mortality rate^1,2^. The incidence of HCC is very high in East Asia (South Korea, China, and Vietnam) and sub-Saharan Africa (>20 per 100,000) compared to America and Europe (<5 per 100,000)^3^. Major risk factors for HCC include hepatitis B virus and hepatitis C virus infections, chronic alcohol abuse, and liver cirrhosis (LC)^4^. Recently, metabolic diseases such as obesity, type 2 diabetes mellitus (T2DM), and non-alcoholic fatty liver disease were proposed to be associated with the development of HCC, raising its risk by 1.5–2.0 fold^5–7^.

After recognition of the link between T2DM and HCC, many studies investigated the effects of anti-diabetic agents on incident HCC. The use of metformin significantly decreased HCC risk by 0.5–0.8 fold^8–10^, probably by ameliorating insulin resistance and inhibiting hepatoma cell proliferation^8^. The effects of thiazolidinedione on HCC development remain controversial^9–11^. In contrast, insulin and oral insulin secretagogues, including sulfonylureas and glinide, markedly increased HCC risk by 1.3–2.6 fold^10,12^, likely by elevating serum insulin concentrations, which promote cancer cell proliferation, invasion, and metastasis^13^. Regarding the different types of sulfonylureas, we previously showed that gliclazide use was associated with a lower risk of HCC compared to glimepiride use^14^. In this study, we investigated the effects of different types of sulfonylureas on HCC development by extending the study’s scope to an examination of nationwide, nested, case-control data from a Korean cohort.

## Results

**Figure 1:**
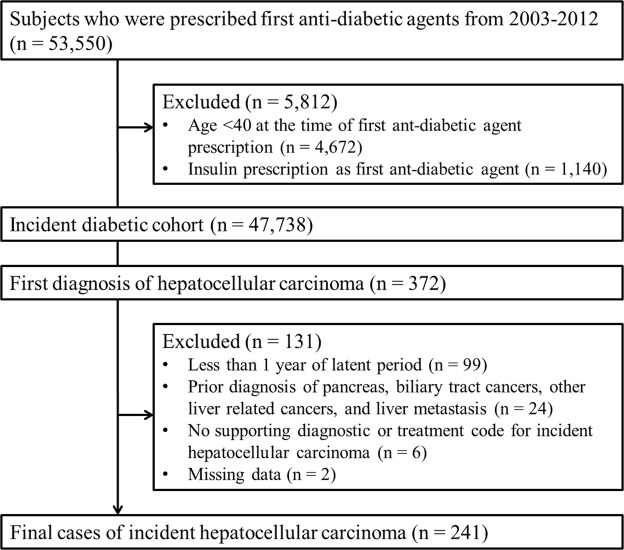
Study flowchart and patient dispositions.

From the study cohort of patients with new-onset diabetes (n = 47,738), 372 subjects with a first-time diagnosis of HCC were identified. After excluding those who did not meet the inclusion criteria (n = 131), 241 final HCC cases and 1,205 matched controls were included in the study (Fig. 1).

The baseline characteristics of participants are shown in Table 1. As case and control patients are matched by age, sex, and year of diabetes diagnosis, these variables were evenly distributed between groups. More than 50% of subjects were over 60 years of age, and most subjects diagnosed with HCC were male. The HCC case group included more patients with chronic viral hepatitis (OR = 26.1, 95% CI = 14.8–26.3), LC (OR = 45.6, 95% CI = 23.7–87.5), and a previous cancer history (OR = 2.37, 95% CI = 1.33–4.23) compared to the control group. In addition, there were significantly fewer aspirin and statin users among the HCC case patients (OR = 0.57, 95% CI = 0.41–0.79 and OR = 0.25, 95% CI = 0.17–0.36, respectively). Among the anti-diabetic agents used, sulfonylurea and glinide use was significantly higher in the HCC group (OR = 1.84, 95% CI = 1.19–2.86 and OR = 2.00, 95% CI = 1.29–3.08, respectively) than in the control group, whereas metformin use was lower in the HCC group than in the control group (OR = 0.59, 95% CI = 0.43–0.81).

**Table 1:** Baseline characteristics of HCC cases and matched controls.

Characteristics	Cases (n = 241) n (%)	Controls (n = 1,205) n (%)	Crude OR (95% CI)
Age at index date, years*			—
40–49	17 (7.1)	101 (8.4)	—
50–59	73 (30.3)	360 (29.9)	—
60–69	82 (34.0)	398 (33.0)	—
70–79	57 (23.7)	297 (24.6)	—
≥80	12 (5.0)	49 (4.1)	—
Sex*			—
Male	194 (80.5)	970 (80.5)	—
Female	47 (19.5)	235 (19.5)	—
Year at diagnosis of diabetes*			—
2003–2006	148 (61.4)	731 (60.7)	—
2007–2009	69 (28.6)	357 (39.6)	—
2010–2012	24 (10.0)	117 (9.7)	—
Alcoholic liver disease	29 (12.0)	100 (8.3)	1.54 (0.98–2.41)
Chronic viral hepatitis	76 (31.5)	19 (1.6)	26.1 (14.8–46.3)
Liver cirrhosis	96 (39.8)	19 (1.6)	45.6 (23.7–87.5)
Chronic respiratory disease	63 (26.1)	310 (25.7)	1.02 (0.74–1.41)
Previous cancer	18 (7.5)	40 (3.3)	2.37 (1.33–4.23)
CCI, mean (SD)	0.99 (1.19)	1.11 (1.24)	0.91 (0.80–1.03)
Household income, mean (SD)	5.54 (3.03)	6.05 (3.21)	0.95 (0.91–0.99)
Residential area			
Metropolitan	106 (44.0)	551 (45.7)	1.00
Non-metropolitan	135 (56.0)	654 (54.3)	1.07 (0.81–1.42)
Aspirin use	59 (24.5)	431 (35.8)	0.57 (0.41–0.79)
Statin use	39 (16.2)	507 (42.1)	0.25 (0.17–0.36)
Insulin use	11 (4.6)	31 (2.6)	1.81 (0.90–3.66)
Sulfonylurea use	212 (88.0)	976 (81.0)	1.84 (1.19–2.86)
Glinide use	33 (13.7)	91 (7.6)	2.00 (1.29–3.08)
Metformin use	162 (67.2)	924 (76.7)	0.59 (0.43–0.81)
Thiazolidinedione use	33 (13.7)	180 (14.9)	0.90 (0.60–1.36)
DPP4 inhibitor use	14 (5.8)	116 (9.6)	0.55 (0.30–1.00)

Abbreviations: HCC, hepatocellular carcinoma; OR, odds ratio; CI, confidence interval; CCI, Charlson comorbidity index; SD, standard deviation; DPP4, dipeptidyl peptidase-4.

*Matching variables. The index date was defined as the year before the diagnosis of HCC.

**Figure 2:**
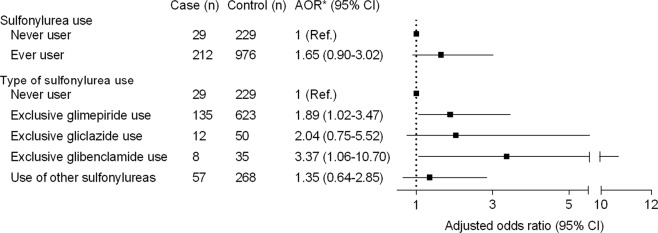
Risk of hepatocellular carcinoma according to sulfonylurea use. Abbreviations: AOR, adjusted odds ratio; CI, confidence interval. *Adjusted for alcoholic liver disease, chronic viral hepatitis, liver cirrhosis, chronic lower respiratory disease, history of previous cancer, Charlson comorbidity index, household income level, residential area, aspirin use, statin use, and other anti-diabetic agent (insulin, glinide, metformin, thiazolidinedione, dipeptidyl depeptidase-4 inhibitor) use.

Figure 2 shows the relationship between sulfonylurea use and HCC. Those prescribed a sulfonylurea had an increased risk of HCC compared to never users, although the association was attenuated after adjusting for comorbidities (alcoholic liver disease, chronic viral hepatitis, LC, chronic lower respiratory disease, previous cancer, and Charlson comorbidity index), household income level, residential area, aspirin use, statin use, and other anti-diabetic agent (insulin, glinide, metformin, thiazolidinedione, and DPP4 inhibitor) use (AOR = 1.65, 95% CI = 0.90–3.02). An analysis of the individual sulfonylureas was performed and the exclusive use of glimepiride or glibenclamide resulted in a significantly increased risk of HCC (AOR = 1.89, 95% CI = 1.02–3.47 and AOR = 3.37, 95% CI = 1.06–10.70, respectively) compared to never users. No statistically significant association was observed between exclusive gliclazide use and incident HCC (OR = 2.04, 95% CI = 0.75–5.52). To evaluate whether there is a dose-response relationship between sulfonylurea use and the rate of HCC, we stratified cumulative duration as cumulative defined daily doses (cDDDs) less than or equal to 180, between 180 and 365, and greater than 365. However, there were no associations among any cDDDs of glimepiride, gliclazide, or glibenclamide (data not shown).

## Discussion

In this nationwide, nested, case-control study, we analyzed 241 HCC cases and 1,205 matched controls at a 1:5 ratio among 47,738 patients with new-onset diabetes from the NHIS-NSC, which included 11 years of follow-up data for 1,025,340 individuals over 40 years of age. We showed that the use of a sulfonylurea tends to increase the risk of incident HCC by 1.7-fold after adjusting for potential confounders, but there was no statistical significance. Regarding the different types of sulfonylureas, glimepiride use significantly increased the risk of HCC compared to never users, and no statistically significant associations were observed between gliclazide use and incident HCC.

Sulfonylureas commonly stimulate insulin secretion by closing ATP-sensitive potassium channels in pancreatic β cells^15,16^. However, each sulfonylurea has slightly different pharmacological properties^17^. In addition to glycemic effects, gliclazide possesses free radical-scavenging activity and upregulates antioxidant enzymes, probably because of its unique molecular structure, which contains an azabicyclo-octyl ring^18^. An experimental study demonstrated that gliclazide can repair DNA damage in mouse insulinoma cells^19^ and attenuate DNA damage and inflammation in T2DM patients^20,21^, suggesting that gliclazide may protect against oxidative stress-related complications, including cancer.

Previous studies have shown the effects of sulfonylureas on HCC. These effects were primarily because of the results of the drug class as a whole^22–24^ and comparisons of the different sulfonylurea agents have led to inconsistent results^12,14,25^. A study in Taiwan showed that the use of first- or second-generation sulfonylureas (e.g., gliclazide, glibenclamide), but not third-generation sulfonylureas (e.g., glimepiride), was associated with incident liver cancer^12^. On the other hand, Monami *et al*.^25^ showed that exposure to gliclazide for at least 36 months significantly reduced incident cancer risk by 0.4 fold, whereas exposure to glibenclamide increased incident cancer risk by 2.6 fold, compared to never users. Our previous study revealed that treatment with gliclazide for more than 2 years was associated with a significantly lower risk of HCC by 0.3 fold compared to those treated with glimepiride^14^. Differences in study designs, sample sizes, and reference groups might explain these conflicting results.

In the present study, we compared the development of HCC over time according to the different types of sulfonylureas using a nationwide, nested, case-control database. Among 241 HCC cases, more than 50% of sulfonylurea users were treated exclusively with glimepiride and we noted that the exclusive use of glimepiride was significantly associated with an increased risk of HCC compared to never users. The use of glibenclamide showed a significantly higher AOR, but the number of subjects was too small to yield clinically meaningful results. Regarding the association between gliclazide use and incident HCC, the risk of HCC tended to increase but was not statistically significant. About 23% of sulfonylurea users were treated with more than two types of sulfonylureas; therefore, we were unable to observe individual drug effects in these subjects.

There are several limitations of our study. First, although we analyzed data from a nationwide sample cohort, the final numbers of case patients and those who had been exclusively treated with gliclazide or glibenclamide were relatively small. We restricted the study population to newly diagnosed T2DM patients and excluded those who had developed HCC less than 1 year after the T2DM diagnosis to assess the pure effects of the sulfonylureas. These factors might further reduce the final number of case patients. Second, information about smoking and alcohol habits, body mass index, and laboratory parameters, such as fasting plasma glucose and glycated hemoglobin levels, was unavailable. Thus, we presumed that diagnosis of chronic lower respiratory disease and alcoholic liver disease represent the smoking and alcohol statuses, respectively. Despite these limitations, we used the established NHIS-NSC database, in which patients have a high prevalence and incidence of HCC. We also chose a nested, case-control model to minimize selection and recall biases compared to traditional case-control studies^26^. Moreover, we strictly defined the inclusion and exclusion criteria to include the new-onset T2DM cohort and incident HCC cases, and results were obtained after adjusting for potential confounders. These restrictions provide more sophisticated results to identify the associations between individual sulfonylureas and HCC incidence in the present study.

In conclusion, the present study showed that those prescribed a sulfonylurea had an increased risk of HCC, which differed by the type of sulfonylurea used. Further longitudinal studies including a larger sample size are warranted to confirm these results and translate them into clinical practice.

## Methods

### Data source

We used data from the National Health Insurance Service-National Sample Cohort (NHIS-NSC) database that were collected from 2002 to 2013 in South Korea^27^. The NHIS-NSC is an established population-based cohort, which represents 2.2% of the entire Korean population. A total of 1,025,340 individuals were randomly selected in 2002 and followed for 11 years, with 1,014,730 individuals remaining in 2013. The database contains information about personal information (age, sex, residential area, income, etc.), medical treatment (diagnosis codes from the 10^th^ revision of the International Classification of Disease [ICD-10] and details of prescription), and general health examination data (physical examinations, laboratory test results, and answers to a questionnaire about lifestyle behaviors). This study was approved by the Institutional Review Board of Severance Hospital, Yonsei University College of Medicine (No. 4-2018-0723). All methods were carried out in accordance with relevant guidelines and the requirement for informed consent was waived because the study used non-identified data.

### Study cohort and case-control patient selection

Among subjects who had not been prescribed any anti-diabetic agent in 2002, we selected subjects who were first prescribed an anti-diabetic agent from 2003 to 2012 (n = 53,550). We excluded patients who were under 40 years of age when first prescribed an anti-diabetic agent (n = 4,672) and were prescribed insulin as the first anti-diabetic agent (n = 1,140). Thus, patients with type 1 diabetes mellitus or underlying T2DM were excluded, resulting in the inclusion of 47,738 new-onset diabetes patients age 40 years or older. Entry into the study cohort was defined as the first anti-diabetic agent prescription date and patients were followed until either they received an HCC diagnosis or December 31, 2013, whichever came first.

Within the study cohort, case patients were included when they were first diagnosed with HCC (ICD-10 code C22.0) with at least a 5-year HCC-free period (n = 372). Exclusion criteria were as follows: (i) subjects who developed HCC less than 1 year after the T2DM diagnosis, (ii) a prior diagnosis of pancreas or biliary tract cancer (ICD-10 codes C23–C25), other liver-related cancers (C22.1–C22.4), or cancers likely to metastasize to the liver (C16, C18–C20, C34, and C50), (iii) not having a clinical diagnostic or treatment code for HCC, and (iv) missing data. Finally, 241 patients were identified as case patients. Control patients were randomly selected from the study cohort at a 5:1 ratio after matching the age at the index date (defined as the date 1 year before the HCC diagnosis), sex, and the year of the diabetes diagnosis with the case group.

### Use of sulfonylureas

Prescription details obtained before the index date were used to define drug use; the use of sulfonylureas was identified as at least one prescription between entry to the study cohort and the index date. The same definition was applied to other drugs, including non-anti-diabetic drugs. In addition, study patients were divided into five groups according to sulfonylurea use: never user, exclusive glimepiride user, exclusive gliclazide user, exclusive glibenclamide user, and user of another sulfonylurea, including nonexclusive glimepiride, gliclazide, or glibenclamide use.

### Statistical analysis

Baseline characteristics of HCC case and control patients are presented as numbers (percentages). To estimate the relationship between sulfonylurea use and HCC risk, conditional logistic regression analysis was performed to estimate the odds ratios (ORs) and 95% confidence intervals (95% CIs). An adjusted OR (AOR) was calculated after adjusting for alcoholic liver disease (K70), chronic viral hepatitis (B18), LC and related complications (K74, K72.1, K72.9, K76.5-K76.7, I85, I86.4, I98.2), chronic lower respiratory disease (J40-J47), history of previous cancer, and Charlson comorbidity index. We also adjusted for household income level, residential area, aspirin use, statin use, and other anti-diabetic agent (insulin, glinide, metformin, thiazolidinedione, and dipeptidyl depeptidase-4 [DPP4] inhibitor) use. As the information on smoking and alcohol habits could not be obtained from the NHIS-NSC data, we used chronic lower respiratory disease and alcoholic liver disease as surrogate variables for smoking and alcohol statuses, respectively. SAS (version 9.4, SAS Institute Inc., Cary, NC) was used for statistical analyses and a *P* value less than 0.05 was considered significant.
